# A Metabolomic Study to Identify Potential Tissue Biomarkers for Indomethacin-Induced Gastric Ulcer in Rats

**Published:** 2019

**Authors:** Reyhaneh Farrokhi Yekta, Nasrin Amiri-Dashatan, Mehdi Koushki, Masoomeh Dadpay, Fatemeh Goshadrou

**Affiliations:** 1. Proteomics Research Center, Shahid Beheshti University of Medical Sciences, Tehran, Iran; 2. Student Research Committee, Faculty of Paramedical Sciences, Shahid Beheshti University of Medical Sciences, Tehran, Iran; 3. Department of Clinical Biochemistry, Faculty of Medicine, Tehran University of Medical Sciences, Tehran, Iran; 4. Department of Pathology, AJA University of Medical Sciences, Tehran, Iran; 5. Department of Basic Sciences, Faculty of Paramedical Sciences, Shahid Beheshti University of Medical Sciences, Tehran, Iran

**Keywords:** Gastric ulcer, Indomethacin, Metabolomics, Nuclear magnetic resonance

## Abstract

**Background:**

Gastric Ulcer (GU) is the most prevalent gastrointestinal disorder induced by various factors and Non-Steroid Anti-Inflammatory Drugs (NSAIDs) as one of the most common reasons. Due to the absence of appropriate molecular markers for GU, the aim of this study was to utilize a metabolomics approach in order to find potential metabolite markers for the disease.

**Methods:**

Stomach tissue samples from indomethacin-treated rats and normal controls were used to perform a 1H-NMR metabolomics study. The altered metabolites were identified using random forest multivariate analysis.

**Results:**

ROC curves showed that the random forest model had a good predictive performance with AUC of 1 for the test and 0.708 for the training sets. Seventeen differentially expressed metabolites were found between GU and normal tissue sample. These metabolites included trimethylamine, betaine, carnitine, methionine, acetylcho line, choline, N,N-Dimethylglycine, cis-aconitate, tryptophan, spermidine, acetylcarnitine, creatinine, pantothenate, taurine, isoleucine, glucose and kynurenine.

**Conclusion:**

The results of the study demonstrated that metabolomics approach could serve as a viable method to find potential markers for GU. Surely, further studies are needed for the validation of the results.

## Introduction

Gastric ulceration is a benign lesion on the mucosal epithelium upon exposure of the stomach to excess acid and aggressive pepsin activity 1. Gastric Ulcer (GU) is a very common gastrointestinal disease which may lead to dangerous complications and even death. It is accounting for an estimated 15 mortality out of every 15,000 complications yearly in the world 2. GU affects approximately 10% of the population worldwide 3, so its prevention and management are considered very important challenges. As a multifactorial disease, it mainly occurs due to imbalance between acid secretion and cytoprotective factors such as bicarbonate secretion, prostaglandins, cell renewal and antioxidants 4. Main factors causing GU usually include *Helicobacter pylori* (*H. pylori*) infection, acid secretion, types of diet, alcohol consumption and Non-Steroid Anti-Inflammatory Drugs (NSAIDs) 5,6. Specifically, gastrointestinal toxicity of NSAID drugs origin may be as high as 4–8% per year and the complications are even higher for those with prior history of ulcer disease 7. NSAIDs are from the most commonly used drugs in the world 8 and NSAID-induced gastric damage is known to be the most common side-effect of these drugs in about 25% of the users 9. Indomethacin (INDO) as the main NSAID is an indole derivative, non-steroidal, inflammatory drug with anti-inflammatory, analgesic, and antipyretic effects 10. It has also been demonstrated to have a stronger effect to induce gastric injury than other currently used NSAIDs. Therefore, indomethacin became the first-choice drug to produce an experimental ulcer model as a result of having higher ulcerogenic potential than other NSAIDs 11. Inhibition of prostaglandin synthesis is one of the mechanisms suggested for the GU caused by NSAIDs 12. It has been suggested that indo-methacin induces gastric damage *via* inhibiting the release of protective factors like COX-1, PGE2, bicarbonate, and mucus; the aggressive factors like acid, and oxidant parameters increase while antioxidant parameters are decreasing 11. Currently, the NSAIDs side effects are only detectable by endoscopy, and no bio-markers have been yet presented. Furthermore, identifying novel biomarkers would likely improve the safety of NSAIDs use. According to the literature, the relation between peptic ulcer and stomach cancer has long been disputed and there is accumulating evidence that gastric ulcer disease is positively associated with the risk of developing stomach cancer. Therefore, identification of high-confidence diagnostic biomarkers is very important for GU. In recent years, many attempts have been made in order to find molecular markers for GU and some candidate biomarkers were also introduced. According to Takeuchi *et al*, hydroxyproline can be a new serum biomarker of gastric injury 13. The results of other studies on serum showed that the NS-AIDs induced decrement of citrate, cis-aconitate, succinate, 3-hydroxy butanoic acid, o-acetyl carnitine, proline and hydroxyproline 14.

“Omics” approaches including genomics, proteomics, and metabolomics have gained much attention in recent years to find potential markers for GU using various biological samples such as tissues, biological fluids and cell cultures. By detection and quantification of all metabolites in a specific sample, metabolomics provides a “snapshot” of metabolic changes related to the disease. Due to more dynamical status of metabolomics rather than both of genomics and proteomics analyses, this approach can detect metabolic changes associated with different physiological states in a shorter time frame. Moreover, metabolomics has the ability to detect and introduce biomarkers in a broad range of samples including whole blood, serum, plasma, urine, saliva and tissues in various disease conditions 15. In recent years, a few studies on gastric ulcer biomarker detection were done by different metabolomics-based techniques in urine and serum samples 16–18. So, in the current study, a nuclear magnetic resonance-based metabolomics approach was investigated to find potential metabolite markers in stomach tissue samples of indomethacin-induced rat models of GU and also to better understand the underlying mechanisms of NSAIDs-induced gastric ulcer.

## Materials and Methods

### Experimental animals

A total of 24 male Wistar rats aging 6–8 weeks with the average weight of 180–220 *g* were used in the study. Rats were kept in temperature controlled houses with a 12 *hr*/12 *hr* dark/light cycles. They were also provided with sufficient water and food access. The rats were kept in houses with raised floors to avoid coprophagy. The rats were randomly divided into 3 groups: group 1=normal rats receiving water (n=8), group 2=indomethacin-induced ulcer rats (n=8), and group 3=rats receiving vehicle (n=8). The rats were fasted for 24 *hr* before the indomethacin administration with free access to water. After that, 45 *mg/kg* indo-methacin was administered 19–21. The water, CMC, and indomethacin were administered by oral gavage to each rat. After 6 *hr*, the animals were anesthetized by 60 *mg/kg* ketamine and 20 *mg/kg* xylazine. The rats were then sacrificed and their stomachs were removed and photographed.

### Ulcer index measurement

The number of ulcers in each stomach were counted and averaged to calculate the ulcer index number according to the following formulae: Ulcer Index= (U/N) ×100, where U is the number of ulcers in the stomachs of group 2 rats and N is the number of rats in this group 22. This study was carried out in accordance with the Guidance for the Care and Use of Laboratory Animals of the NIH. The experiment was approved by the clinical ethics committee of Shahid Beheshti University of Medical Sciences.

### Histopathology analyses

The rats’ stomachs were opened along the greater curvature and were completely washed with normal saline to remove any contaminants. A part of the stomach samples was flash frozen with liquid nitrogen and stored at −80*°C* for metabolomics analysis. The other part of the stomach samples was fixed in 10% formalin and was paraffin embedded to pathologically confirm the gastric ulcer in rats. The paraffin embedded samples were cut into 5 *μm* thick sections and stained with Hematoxylin and Eosin (H&E) solution to microscopically determine the ulcer regions by pathologist.

### Sample preparation for metabolomics study

For preparation of the stomach tissue extracts, 300 *mg* of the frozen tissues were grounded completely in liquid nitrogen and homogenized in 1 *ml* of 2:1 *v/v* Methanol/Chloroform solution. After that, 1 *ml* of 1:1 *v/v* Chloroform/H_2_O was added and the solution was centrifuged for 20 *min* at 15,000 *g* and 4*°C* 16. 600 *μl* of the upper phase was then collected and lyophilized. For Nuclear Magnetic Resonance (NMR) analysis, the lyophilized tissue extract was dissolved in 600 *μl* of phosphate buffer solution containing 80% D_2_O, 2% TSP (trimethylsilyl propionate), 4% KH_2_PO_4_ and 0.01% NaN_3_.

### 1H-NMR spectrometry

The 1H-NMR analysis was performed on a Bruker Avance 400 *MHz* instrument equipped with 5 *mm* probe at 298 *K*. The Carr-Purcell-Meiboom-Gill (CPMG) platform was used by a standard pulse sequence irradiating residual water peak, relaxation delay of 2 *s* and total T2 relaxation time of 60 *ms*. Other features of the spectrum collection included 150 total scans, spectral width of 8389.26 *Hz*, 90^°^ pulse width and 0.5 *Hz* line broadening prior to Fourier transformation. The spectra were phased and base-line corrected and were referenced to the peak of TSP at 0 *ppm*. The NMR spectra were binned in the range of 0.3 and 9.5 as 0.01 *ppm* parts and were normalized and log-transformed. The region between 4.5 and 5.5 *ppm* was also omitted for water signal suppression. The NMR spectra were deconvoluted by ProMetab software in MATLAB.

### Statistical analyses

The data matrix resulted from 1H-NMR analysis was used to perform multivariate statistical modeling to identify the most significant and relevant metabolites differentiating gastric ulcer from normal controls. The Random Forest (RF) algorithm was implemented using MATLAB software. Random Forest is a machine learning method based on the construction of multiple decision trees by bootstrapping the data 23. Each decision tree predicts an independent classification of the samples. The original dataset resulted from 1H-NMR analysis was divided into training and test sets. About one third of the samples did not participate in the construction of the model which are called Out Of Bag (OOB). After construction of the model, each OOB is entered to its relevant kth decision tree to estimate the classification ability of the RF model. The predictive performance of the RF model was measured based on the difference between the predicted and expected outcomes by counting the number of True Negatives and Positives (TN, TP) and False Negatives and Positives (FN, FP). The following formula were used to assess sensitivity, specificity, precision (positive predictive value), accuracy and overall error rate: sensitivity= TP/TP+FN, specificity= TN/TN+FP, precision (PPV)= TP/TP+FP, accuracy= TP+TN/P+N, overall error rate= (FP+FN/P+N)×100, where P and N indicate the total number of positive and negative values, respectively. The predictive power of the model was demonstrated by Receiver Operating Characteristic (ROC) Curves for both training and test datasets.

### Metabolites identification and pathway analysis

The variables (NMR spectral bins) with the highest importance value resulted from the RF model which had p-values of less than 0.05 were considered significant. The metabolites were identified using relevant databases of NMR metabolomics including Biological Magnetic Resonance Bank (BMRB) 24 and Human Metabolome Database (HMDB) 25. The tolerance for searching spectral bins was ±0.01 *ppm*. The significantly altered metabolites were then used to find the most important pathways in the pathogenesis of gastric ulcer. The pathway enrichment analysis was performed using the MetaboAnalyst 4 26 online server.

## Results

**Figure 1.**
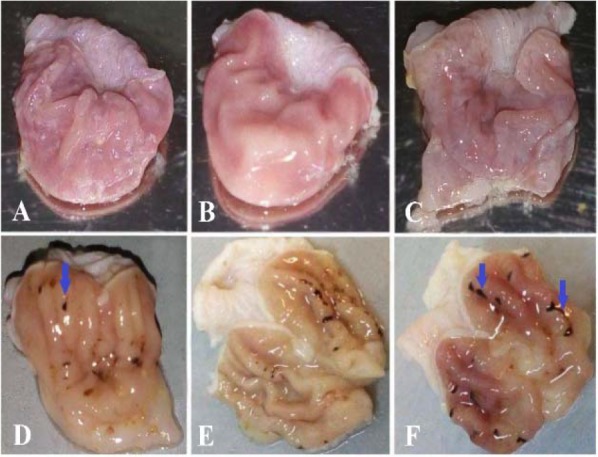
The macroscopic appearance of the stomach from (A, B) normal control, (C) control receiving CMC and (D–F) indomethacin-induced gastric ulcer rats. Arrows show linear and focal hemorrhagic areas.

**Figure 2.**
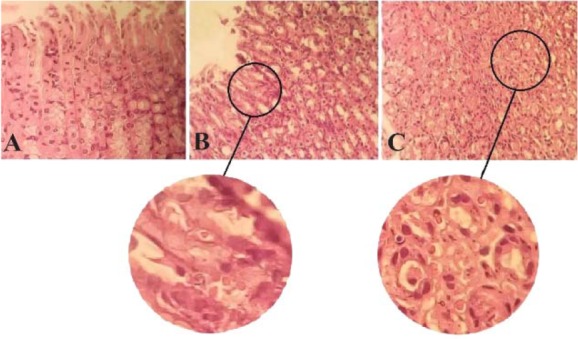
The gastric mucosa appearance in (A) normal and (B, C) indomethacin-induced lesions stained with H&E (100x magnified). Normal stomachs have intact epithelium with distinct chief and parietal cells where ulcer areas show epithelium damage and infiltration of lymphocytes and monocytes.

**Figure 3.**
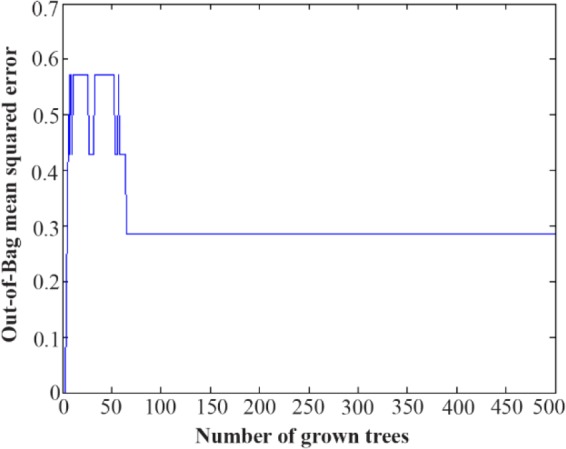
The plot of the OOB error for the random forest model.

**Figure 4.**
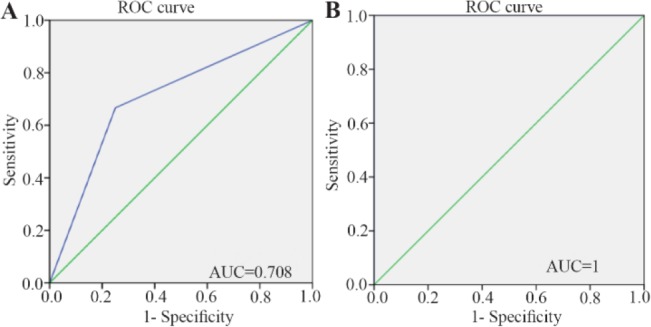
ROC curves demonstrating predictive performance of the RF model for (A) training and (B) test sets.

**Figure 5.**
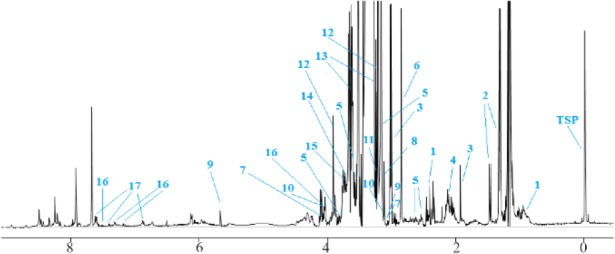
The representative 400 *MHz* CPMG 1H-NMR spectrum of rat stomach tissue. Altered metabolites between normal and indo-methacin-induced gastric ulcer samples are demonstrated. Key: 1-pantothenate, 2-isoleucine, 3-spermidine, 4-methionine, 5-acetylcarnitine, 6-trimethylamine, 7-creatinine, 8-carnitine, 9-cisaconitate, 10-choline, 11-taurine, 12-betaine, 13-glucose, 14-N,NDimethylglycine, 15-acetylcholine, 16-tryptophan, 17-kynurenine.

**Figure 6.**
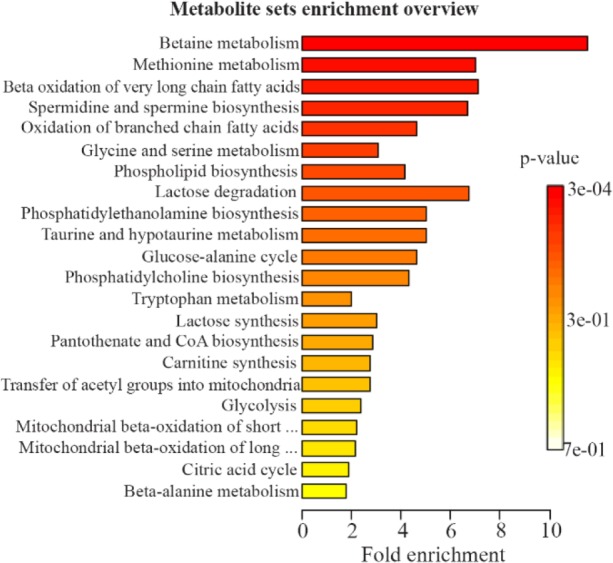
The metabolite sets enrichment analysis (MSEA) overview.

The stomach tissues of the rats in each group were utilized for a metabolomics investigation to find potential diagnostic tissue markers for gastric ulcer. In this study, an attempt was made to compare 3 groups including normal controls which only received drinking water, rats with GU induced by indomethacin, and the third group which received CMC as vehicle. The macroscopic view of the stomachs with ulcer showed areas with linear and focal hemorrhage (Figure 1). The sections of the gastric mucosa of the indomethacin-treated group also demonstrated gastric erosion and infiltration of inflammatory cells and leukocytes compared to control group (Figure 2). 1H-NMR metabolomics study was performed and the most significant metabolites were identified using the multivariate random forest analysis. The RF model resulted in important values for the variables, where larger values denoted for the most important variables in the discrimination of normal and ulcer groups. The predictive performance of the model was assessed by ROC curve and the Area Under Curves (AUC). The AUC represents the diagnostic potential of the RF model and the predictive behavior of the classifier. A perfect prediction yields in an area under curve of 1 where the diagonal line shows a random prediction result with AUC of 0.5. The AUC for the training set was 0.708 where this value was 1 for the test set. The ROC curves are shown in figure 3 and the RF model performance metrics are shown in table 1. The OOB error rate was also measured for the model as can be seen in figure 4. The metabolome analysis of groups 1 and 3 did not show any significant differences where the altered metabolites between groups 1 and 2 were considered as potential markers for gastric ulcer. These metabolites included over- expression of trimethylamine, carnitine and acetylcholine, and down-regulation of choline, N,N-Dimethyl-glycine, cis-aconitate, tryptophan, spermidine, acetylcarnitine, creatinine, pantothenate, taurine, isoleucine, betaine, methionine, glucose and kynurenine (Figure 5). The complete details on the altered metabolites are demonstrated in table 2. The metabolite sets enrichment analysis revealed that the most important pathways involved in the pathogenesis of gastric ulcer included betaine metabolism, methionine metabolism, beta-oxidation of very long chain fatty acids and spermidine and spermine biosynthesis (Figure 6, Table 3).

**Table 1.** The RF model predictive performance features

	**Overall error rate**	**Sensitivity**	**Specificity**	**Accuracy**	**Precision**
**Test set**	0	100%	100%	100%	100%
**Train set**	28.60%	66.70%	75%	71.42%	66.70%

**Table 2.** The significantly altered metabolites between GU and normal control

**Metabolite**	**Chemical shifts (δ)**	**KEGG ID**	**RF model importance**	**p-value**	**Fold change (ulcer/control)**
**Choline**	3.195, 3.505, 4.055, 3.515	C00114	0.0331	0.0172	3.28 ↓
**Cis-aconitate**	3.095, 3.105	C00417	0.0263	2.32E-05	2.05 ↓
**Tryptophan**	3.285, 3.295, 3.465, 3.475	C00078	0.0240	0.0066	3.79 ↓
**Spermidine**	3.155, 3.145	C00315	0.0194	0.0010	6.40 ↓
**Trimethylamine**	3.255	C00565	0.0190	0.0276	1.75 ↑
**N,N-Dimethylglycine**	2.915, 3.705	C01026	0.0190	0.0150	2.50 ↓
**Acetylcarnitine**	3.175, 3.605, 3.185, 3.595	C02571	0.0138	0.0184	1.90 ↓
**Creatinine**	4.045	C00791	0.0138	0.0157	6.60 ↓
**Pantothenate**	3.425	C00864	0.0135	0.0085	3.52 ↓
**Betaine**	3.265	C00719	0.0125	0.0490	1.60 ↓
**Taurine**	3.405, 3.415, 3.395, 3.385	C00245	0.0125	0.0010	7.90 ↓
**Carnitine**	3.205, 3.215	C00318	0.0123	0.0128	1.80 ↑
**Isoleucine**	3.655, 3.665	C00407	0.0121	0.0061	5.05 ↓
**Glucose**	3.455, 3.235, 3.525, 3.725, 3.825	C00031	0.0119	0.0010	3.88 ↓
**Kynurenine**	3.695	C00328	0.0114	0.0113	5.50 ↓
**Methionine**	2.115, 3.855	C00073	0.0110	0.0447	2.18 ↓
**Acetylcholine**	3.205	C01996	0.0057	0.0285	2.00 ↑

**Table 3.** The significant biochemical pathways involved in the pathogenesis of gastric ulcer

**Pathway**	**Matched metabolites**	**p-value**	**FDR**
**Betaine metabolism**	Betaine, Dimethylglycine, Choline, Methionine	2.62E-4	0.0216
**Methionine metabolism**	Betaine, Dimethylglycine, Choline, Methionine, Spermidine	4.37E-4	0.0216
**Beta-oxidation of very long chain fatty acids**	Carnitine, Acetylcarnitine	0.0304	0.839
**Spermidine and spermine biosynthesis**	Methionine, Spermidine	0.0339	0.839

## Discussion

Gastric ulcer is the upper gastrointestinal mucosa damage caused by helicobacter pylori and NSAIDs such as indomethacin as major reasons. 1H-NMR spectroscopy is a very powerful tool for profiling and comparison of tissue samples metabolic profiles with some advantages over other techniques such as easier sample preparation, the high reproducibility and lower costs. However, a few studies evaluated the stomach tissue metabolic alterations induced by indomethacin using metabolomics analysis to better understand disease mechanism, drug toxicity, drug response and to distinguish predictive biomarkers 17,27. In the current study, metabolite comparison in stomach tissue samples of control and indomethacin treated group was performed. According to the study results, betaine decreased in treated group. Betaine (trimethylglycine) is known as an antioxidant in previous reports. Based on Alirezaei *et al*, lipid peroxidation significantly decreased in betaine pretreated rats and significantly decreased ulcer occurrence 28. Alterations of betaine content was previously observed in rat models of gastric ulcer 29. Methionine and isoleucine amino acids decreased in our study. Methionine is an essential amino acid in humans which is a substrate of other amino acids such as taurine and also the important antioxidant, glutathione. Previous studies reported that some of amino acids including methionine and leucine inhibit indomethacin-induced gastric ulcers at a dose-dependent manner 30. According to the present study results, decreased levels of these amino acids in the stomach susceptible to the ulceration depleted their protective function.

In this study, taurine level was significantly decreased in rats treated with indomethacin. Taurine is an intracellular free thiol-containing β-amino acid that can be found in various mammalian tissues. It has been reported that taurine plays important biological roles including nutrition, antioxidation, anti-inflammatory function, membrane stabilization, modulation of intra-cellular free calcium concentration and protection against oxidant-mediated injury in several organs 31,32. Indeed, it protects against the drug-related gastric damage and colonic injury by its antioxidant properties. Antioxidant function of taurine in membrane organization is done by its protection against free radicals. In addition, the results of several studies have shown that taurine prevents gastric ulcer induced by indomethacin through lipid peroxidation inhibition and neutrophil activation 33,34. Furthermore, taurine changes might imply the oxidative stress-related gastric ulceration. Decreased taurine level was also reported in a metabolomic study by Um *et al* in indomethacin-induced gastric ulcer rat models 29. The result of our study indicates that glucose level decreased in the indomethacin-treated group, indicating excessive glucose was consumed to ameliorate gastric injury. Several investigations have found glucose metabolism is increased in cell transformations 35,36. According to our results, indomethacin administration increased kynurenate and decreased choline and tryptophan levels. Indomethacin stimulated the conversion of tryptophan into kynure-nate, which inhibits fibroblast growth factor and delays ulcer healing. Fibroblast growth factors are major factors in ulcer healing in stomach mucosa by using embryogenesis and tissue regeneration function 37,38. NS-AIDs such as indomethacin inhibit COX-1 and COX-2 which lead to suppression of prostaglandins. FGFs accelerate healing rate through increasing microcirculation around the ulcer and COX-2-derived prostaglandins 39,40. Our finding indicated that the lack of metabolism of choline to produce phosphatidylcholine caused the gastric mucosa damage. All of these findings suggested depleted protective compounds role in the gastric mucosa damage 41,42. Cis-aconitate is another metabolite that decreases in indomethacin-induced ulcer group which occurs as a result of inhibition of aconitase. Aconitase catalyzes citrate to isocitrate *via* cisaconitate in the Tricarboxylic acid cycle (TCA). Because this compound is one of the intermediates in the TCA cycle, this alteration might suggest the disturbance of energy metabolism in GU 42. Alterations of serum cis-aconitate level was previously reported in a metabolomics study of Takeuchi *et al* on gastric ulcer induced by nonsteroid anti-inflammatory drugs 43. Glycine is a glucogenic amino acid and provides glucose for energy metabolism. It was also reported that glycine is essential for defense system in cells and helps in digestion of fats by the bile acid regulation 29,44. N, NDimethylglycine decreased in treated group in our study that could be due to elevated energy consuming to protect against gastric damage 45,46.

In our observations, carnitine and Trimethylamine (TMA) increased and acetylcarnitine decreased in indomethacin-induced gastric ulcer group. Acetyl carnitine is an acetylated form of carnitine that is broken down to carnitine which is used by the body to transport fatty acids into the mitochondria for breakdown. Carnitine is a quaternary amine and an essential cofactor which plays important role in long chain fatty acid oxidation in mitochondria 47. According to numerous studies, it is known that carnitine and its derivatives are the main compounds in prevention of reactive oxygen formation and also has a protective capacity in biological membranes against peroxidative stress. Free radicals and peroxidative stress are involved in gastric mucosal damage pathogenesis and based on previous data, carnitine contains beneficial effects by antiperoxidative function on ethanol-induced gastric mucosal damage 48,49. It has been recently reported that carnitine has gastroprotective effects on indomethacin-induced gastric mucosal injury in rats 50,51. TMA is a common metabolite in animals that is oxidized to trimethylamine -oxide. Increased TMA in the body shows the presence of high carnitine level that is converted to TMA. Jung *et al* found increased levels of TMA in naproxen-treated rats 42. According to our results, acetylcarnitine has low levels due to its conversion to carnitine. Then the carnitine level goes up and finally TMA level also increases. These findings indicated elevated energy metabolism in order to defend against gastric mucosal damage. Alterations of trimethylamine were previously reported in gastric ulcer 29. Spermidine and pantothenate are other decreased metabolites in drug-induced gastric damage group in this study. Spermidine is a polyamine compound found in ribosome and living tissues having various metabolic functions within organisms. Spermidine content in mammalian cells has vital roles in protection of cells from oxidative damages, cell proliferation, differentiation and apoptosis 52. Given the very important role of spermidine in cell survival, its decreased levels in damaged stomach tissue seems logical. Previous investigations demonstrated inhibitory effects of polyamines such as spermidine on gastric ulceration and acid secretion. In another study, the effect of polyamines on gastric ulceration in rats was evaluated 53,54. Subcutaneous or oral administration of spermidine and spermine was shown to have inhibitory effects in stress-induced gastric ulceration 54. In a previous study, decreased levels of a spermidine derivative, N8-acetylspermidine, in serum samples were also observed 55.

The other altered metabolite, pantothenic acid (vitamin B_5_), is an essential nutrient that animals require in order to synthesize coenzyme-A, as well as to synthesize and metabolize proteins, carbohydrates, and fats. Studies on animals showed that metabolic effects of pantothenic acid in humans would be widespread because pantothenic acid is the active principle of coenzyme-A, which may play an important role in the metabolism of parietal cells. On the other hand, tricarboxylic acid cycle is the major pathway of energy production in organisms. Pantothenic acid is of the acetyl-CoA components, thus, alteration of pantothenic acid level could affect acetyl-CoA metabolism and finally might influence TCA cycle 56,57. In our results, pantothenic acid decreased in ulcerated model in comparison with control group, which indicated the down-regulation of energy consumption by the TCA cycle and increased energy consumption by lipid oxidation which suggested that indomethacin influences energy metabolism which led to free radicals production, oxidative stress and stomach tissue ulceration.

## Conclusion

In this study, 1H-NMR metabolomics analysis was performed on stomach tissue samples of indomethacin-induced gastric ulcer rats in order to find putative diagnostic biomarkers between control and NSAID-induced gastric ulcer group. The results of this study demonstrated that metabolomics-based investigations can be used to effectively identify biomarkers for GU caused by indomethacin treatment. The metabolic differences between rats in control group and rats treated with indomethacin were classified based on the multivariate random forest model. Several putative biomarkers were identified for diagnosis of NSAID-related gastric ulcer including alterations of pantothenate, isoleucine, spermidine, methionine, acetylcarnitine, trimethylamine, creatinine, carnitine, cis-aconitate, choline, taurine, betaine, glucose, N,N-Dimethylglycine, acetylcholine, tryptophan, and kynurenine. In this study, all of the rats were treated with the same dose of indomethacin, but each of rats in a same group showed different ulcer degrees. This difference seems to be justified by the difference in the response to the drug in each of the rats. Despite the identification of several potential metabolite biomarkers in this study, further investigations are needed to clarify identified metabolites and also on the potential role of these metabolites in the disease pathology and consequently in the development of new NSAID drugs. The present study demonstrated that metabolomics can be used as a new, simple and rapid approach to identify molecular biomarkers for NSAID drugs-induced gastric ulcers. Moreover, metabolomics is a powerful tool to determine drug toxicity and biological pathways involved in drug- related gastric damages.
